# Health-Promoting Food Pricing Policies and Decision-Making in Very Remote Aboriginal and Torres Strait Islander Community Stores in Australia

**DOI:** 10.3390/ijerph15122908

**Published:** 2018-12-19

**Authors:** Megan Ferguson, Kerin O'Dea, Jon Altman, Marjory Moodie, Julie Brimblecombe

**Affiliations:** 1 School of Public Health, The University of Queensland, Brisbane 4072, Australia; 2 Wellbeing and Preventable Chronic Diseases, Menzies School of Health Research, Darwin 0811, Australia; julie.brimblecombe@monash.edu; 3 Division of Health Sciences, University of South Australia, Adelaide 5001, Australia; Kerin.O'Dea@unisa.edu.au; 4 Alfred Deakin Institute for Citizenship and Globalisation, Deakin University, Burwood 3125, Australia; jon.altman@anu.edu.au; 5 Deakin Health Economics, Centre for Population Health Research, Deakin University, Geelong 3220, Australia; marj.moodie@deakin.edu.au; 6 Faculty of Medicine, Nursing and Health Sciences, Monash University, Melbourne 3168, Australia

**Keywords:** food security, diet-related chronic disease, policy, food pricing

## Abstract

Aboriginal and Torres Strait Islander people living in remote communities in Australia experience a disproportionate burden of diet-related chronic disease. This occurs in an environment where the cost of store-purchased food is high and cash incomes are low, factors that affect both food insecurity and health outcomes. Aboriginal and Torres Strait Islander storeowners and the retailers who work with them implement local policies with the aim of improving food affordability and health outcomes. This paper describes health-promoting food pricing policies, their alignment with evidence, and the decision-making processes entailed in their development in community stores across very remote Australia. Semi-structured interviews were conducted with a purposive sample of retailers and health professionals identified through the snowball method, September 2015 to October 2016. Data were complemented through review of documents describing food pricing policies. A content analysis of the types and design of policies was undertaken, while the decision-making process was considered through a deductive, thematic analysis. Fifteen retailers and 32 health professionals providing services to stores participated. Subsidies and subsidy/price increase combinations dominated. Magnitude of price changes ranged from 5% to 25% on fruit, vegetables, bottled water, artificially sweetened and sugar sweetened carbonated beverages, and broadly used ‘healthy/essential’ and ‘unhealthy’ food classifications. Feasibility and sustainability were considered during policy development. Greater consideration of acceptability, importance, effectiveness and unintended consequences of policies guided by evidence were deemed important, as were increased involvement of Aboriginal and Torres Strait Islander storeowners and nutritionists in policy development. A range of locally developed health-promoting food pricing policies exist and partially align with research-evidence. The decision-making processes identified offer an opportunity to incorporate evidence, based on consideration of the local context.

## 1. Introduction

Aboriginal and Torres Strait Islander people living in remote areas generally experience the poorest health outcomes and hold the worst economic position in Australia [1,2]. Aboriginal and Torres Strait Islander people experience unemployment at 4.2 times, and have an average disposable income 70% of, non-Indigenous Australians [3]. Poverty is greatest for Aboriginal and Torres Strait Islander people living in very remote areas and is growing [2]. The life expectancy of Aboriginal and Torres Strait Islander people is approximately 10 years less than non-Indigenous Australians. The majority of this gap is due to chronic disease, especially cardiovascular disease and cancer, and injury for the 35–74 years age group [4]. The gap is largest in remote areas where Aboriginal and Torres Strait Islander people experience a burden 2.4 times that of non-Indigenous people [5]. Dietary intake is a key risk factor contributing to this gap [4,5].

Nutrient-rich traditional, non-market food continues to contribute to dietary intake [6], though the rapid nutrition transition resulting from colonization has led to a population diet high in sugar, salt and fat and low intakes of vegetables, fruit and other nutrient-rich foods [7]. In remote Aboriginal and Torres Strait Islander communities, Western foods are predominantly purchased from the single retail food outlet, referred to as the store, operating in a challenging, remote environment, which contributes to the high cost of food. Many stores are community-owned, providing a unique opportunity for local policy development [8].

The remote store landscape has undergone considerable change in the last decade, particularly in policy and services. In 2008, a Close the Gap statement of intent was agreed to by a number of Aboriginal and Torres Strait Islander people and organizations and the Australian Government [9]. In the same year, the Council of Australian Governments released the Closing the Gap Strategy that aimed to achieve health equity within 25 years [10] and in 2009 developed the National Strategy for Food Security in Remote Indigenous Communities which linked food security (i.e., the ability to acquire appropriate and nutritious food in a regular and socially acceptable manner) and nutrition with the national Closing the Gap targets [11]. Two years prior to this in the Northern Territory (NT) of Australia, the Northern Territory Emergency Response was implemented and included a number of measures indirectly related to food ‘security’. One of these was for the compulsory income management of welfare recipients [12] (i.e., restriction of available cash and purchase of specific products), which has since been extended beyond the NT [13]. A second measure was the introduction of a regulatory framework for the operation of remote stores, including minimum standards relating to food security; this remains effective today [14]. The Australian National Audit Office reports however, that government policies have made minimal contribution to addressing food insecurity in remote communities [15]. Reports on the Closing the Gap targets show mixed outcomes, though importantly that the target to close the life expectancy gap is not on track [10] and that outcomes are worse in remote than non-remote areas [2]. The Productivity Commission highlights the importance of developing an evaluation culture in Aboriginal and Torres Strait Islander policy where policy evaluation informs future policy [16].

During this time of policy change there has also been a growth in organizations that provide retail management services to remote community storeowners, alongside an increasing recognition of the role that the stores play in the health of the communities [17,18,19,20,21]. The historical tension between economic and health outcomes may be giving way as organizations publicly demonstrate valuing health outcomes as an objective of sustainable business [19,21,22,23,24,25]. In remote Aboriginal and Torres Strait Islander community stores, there are examples of local policies (i.e., the rules of operation determined by the governing body [26]), which aim to promote health outcomes within a sustainable business model [24,25,27]. There is significant opportunity in this dynamic remote retail context to work with storeowners and the systems they operate within to influence local store food policy to create health-promoting environments.

Food pricing is considered one of the more effective practices to influence consumer purchasing patterns [28]. Health-promoting food pricing policies exist in remote stores, but there is little understanding of the decision-making process informing their design development including the magnitude of the price increase or decrease and promotion of the policy [29]. Policy analysis can help understand the process of design development and thereby identify opportunities to strengthen design and improve health outcomes through the store [30,31]. Policy development models have evolved to consider trade-offs between multiple and often conflicting objectives [32]; they may have utility in understanding efforts in the remote retail context where governing bodies deal with the dual and potentially conflicting objectives of consumer health outcomes alongside commercial viability of stores. Decision-making which incorporates evidence will hopefully lead to consideration of a greater range of policy options and result in more effective outcomes [33].

This paper describes health-promoting food pricing policies including their alignment with evidence, and the decision-making processes in their development in very remote Aboriginal and Torres Strait Islander community stores in Australia. We specifically refer to ‘food policy’ as the local-level food policy implemented in stores aimed at modifying the price of food/beverages in order to promote health.

## 2. Methods

### 2.1. Context

Approximately 175 stores supply food in some of the 1187 discrete Indigenous communities in remote locations across Australia [8,34]. A total of 92,960 Aboriginal and Torres Strait Islander people and a small number of non-Indigenous people reside in these communities. Seventeen communities have a population greater than 1000 and almost 75% are located in very remote locations [34]. Our study included very remote communities only [35]. These are located largely in the NT, Queensland (Qld), South Australia (SA) and Western Australia (WA). Some stores are owned by the government or are privately owned, though the most common model is of incorporated community ownership where Aboriginal and Torres Strait Islander residents comprise the membership. These stores function as either not-for-profit or business enterprises and are often responsive to community priorities. The owners of community-owned stores employ a store manager/s or engage the services of a retail organization to manage the store’s operation, with the latter model accounting for approximately 55% of stores in remote Aboriginal and Torres Strait Islander communities in Australia [17,18,19,20,21]. In addition to operating an effective retail operation, a number of stores and retail organizations aim to employ local Aboriginal and Torres Strait Islander people and promote positive nutrition outcomes and healthy lifestyles [8].

### 2.2. Design

A qualitative study was conducted that applied a methodology informed by Thow’s framework used in the Pacific Region. This framework was informed by policy theories related to lesson drawing to understand the form of food policies and how to engage with policy-makers [30]. It was successfully used to describe the common elements of policy processes across the diversity of policy processes identified in different countries in the Pacific Region. Our methodology was informed by this framework as we similarly anticipated a diversity of policy processes across different remote communities, states and territories and governance models. We first focused on determining the range of pricing policies in place in remote stores and secondly on an understanding the stages of the process [32], the people involved [30,33], identification of objectives [32], consideration of assessment criteria applied [32] including a list of pre-determined criteria previously used in food policy assessment (i.e., feasibility, sustainability, acceptability, importance, effectiveness, unintended consequences [36,37,38,39]), and the evidence considered.

### 2.3. Data

Purposive sampling was employed, informed by the snowball method, to maximize coverage of the types of policies implemented. Participants were: (i) retailers, who were the store managers employed by the owners of a community store or store managers and retail management staff employed by a retail organization, and (ii) health professionals, including public health nutritionists (hereafter, nutritionists) and others working in roles with stores employed by a retail organization, government or non-government organization. Participants were required to identify that they had knowledge of health-promoting food pricing policy in remote Aboriginal and Torres Strait Islander community stores. At least one retailer and where applicable, the nutritionist from each of five retail organizations representing the majority of these entities, and all nutritionists in service provision and food supply policy known to Megan Ferguson and Julie Brimblecombe operating in remote NT, Qld, SA and WA, were invited to participate. Participants were invited by email from the lead researcher or by a potential participant in the study. This study did not seek to quantify policy implementation by store, store governance model or state/territory.

A semi-structured interview guide was used in all interviews. It focused on two sets of data. The first was the health-promoting food pricing policies in stores. We included price increases and subsidies in the form of price discounts, rewards, vouchers and free product give-away. We excluded takeaway food outlets as a setting and government policy instruments that might impact on food purchases such as income management. The second set of data focused on the decision-making process for one of the policies reported. Interviews lasted on average 50 min, and were conducted by Megan Ferguson, a nutritionist who has worked in both the remote health and retail sectors. This background was important in terms of understanding the context and relating to participants’ experiences. Interviews were conducted in English, in person or by phone. In one case, responses to the interview questions were e-mailed by a participant. Participants provided consent and all interviews were audio-recorded, transcribed verbatim and returned to participants for checking. Documents describing food pricing policies were sourced or provided by participants and used to complement interview data. Data were uploaded and managed in NVivo (QSR International Pty Ltd. Version 11, Melbourne, Victoria, Australia, 2012). Ethical approval for this study was provided by Human Research Ethics Committees in the NT (HREC NTDHMSHR 2012–1711; CAHREC HREC-12-13; CDU HREC H12096), Qld (FNQ HREC HREC/16/QCH/35-1041) and WA (WACHS HREC 2016/13; WAAHEC 715; KAHPF 2016-006). Informed consent was obtained from all participants.

### 2.4. Analysis

The dataset was reviewed independently by two researchers, Megan Ferguson and Julie Brimblecombe, who have extensive research, policy and practice experience in the remote retail and health sectors. This strengthened the analysis by ensuring research quality and relevance. The authors discussed and agreed on the coding framework. The data were coded by Megan Ferguson and the findings reviewed with Julie Brimblecombe.

Firstly, a data content analysis relating to the types and design of food pricing policies was conducted, with allowance for additional codes. The coding framework included the following: Under the three pre-determined codes, *subsidy, price increase, subsidy/price increase combination*; the sub-codes relating to each code of *targeted food or beverage, magnitude of price change, duration, administration, complementary strategies, other design elements*; and, a fourth emergent code, *business fundamentals*. Secondly, a deductive, thematic analysis of the decision-making process was conducted to identify why, how and who was involved.

## 3. Results

### 3.1. Participants

Between September 2015 and October 2016, 47 interviews were conducted with 15 retailers, 28 nutritionists and four health professionals servicing communities in NT, Qld, WA and SA. Forty-two more people were invited to participate by Megan Ferguson; two delegated the interview invitation to staff under their supervision, 21 did not respond to the email invitation and 19 declined, with the most common response being that they did not have sufficient knowledge relevant to the study objectives.

### 3.2. Health-Promoting Food Pricing Policies

The most commonly implemented food pricing policies across very remote Australia were subsidies and subsidy/price increase combinations as shown in Table 1. These policies mostly targeted fruit, vegetables, bottled water, artificially sweetened and sugar sweetened carbonated beverages, in addition to groups of foods broadly referred to as ‘healthy/essential’ foods and ‘unhealthy’ foods. Magnitude of price changes ranged from 5% to 25%. The policies were largely ongoing. A number of these, predominantly those targeting fruit, vegetables and ‘healthy/essential’ and ‘unhealthy’ foods, had been in place for many years including in some locations for over 35 years, where the beverage policies were first introduced in 2010. Short-term discounts were applied more recently and were usually up to two weeks duration. Stores generally funded the long-term policies, such as fruit and vegetables discounts, while more recently implemented policies were partly funded by the suppliers and manufacturers. Pricing policies were at times supported by one or more merchandising strategies involving product availability (e.g., specific brand, quality), placement (e.g., shelf space allocation, planograms) and promotion (e.g., in-store announcements, use of local celebrities), though implementation of these strategies seemed more ad hoc than planned. Promotion of the ongoing pricing policies did not occur and was identified as a missed opportunity in communicating the policy to customers.

“I reckon that it is not visible to the average person in terms of what pricing policies stores have … and therefore not as effective. … I don’t think that translates to the customer that they’re getting a good deal on whatever they’re getting a good deal on.”

(Interviewee 47, Health professional)

Finally, retailers and health professionals stressed the requirement of efficient and effective retail operations as the key condition for the development of health-promoting pricing policies.

### 3.3. Decision-Making

#### 3.3.1. Process of Decision-Making

The process of decision-making reported included some level of deliberation and procedure, though this was generally described as flexible. The processes described by those in retail organizations were more structured with specific stages of development, than those described for stores operating independently. However, there were often more people involved in the decision-making processes of retail organizations than in individual community stores.

#### 3.3.2. Decision-Makers

Three groups of people were identified as being involved in policy development, namely retailers, nutritionists employed by retail organizations, and Aboriginal and Torres Strait Islander and non-Indigenous store committee/board members. There were a few cases where nutritionists or health professionals employed by the health sector contributed directly to the process. Retailers and/or nutritionists employed by retail organizations reported that they primarily identified the need for, and designed policies, though the need for a policy was said to be identified sometimes by Aboriginal and Torres Strait Islander storeowners.

“… we are working with (X) communities at the moment to reduce the sale of full sugar soft drink. And I must note the communities or the storeowners approached us about it. So we talk about the health stats every quarter. But now that there’s more education around you know, the impacts of diet and poor health and those things. Now storeowners are saying, ‘What can we do to improve these outcomes?’”

(Interviewee 40, Retailer)

Policies were reported to be approved by the store committee/board, and at times, by retailers. Examples were provided where store committees/boards were reported to actively direct and monitor policy, whereas others provided support or opposition to policy proposals initiated by retailers.

#### 3.3.3. Policy Objectives

Price manipulation was seen by most participants as a means to increase purchases of healthy foods and to reduce purchases of unhealthy foods and hence improve the quality of dietary intake, with participants acknowledging the high rates of overweight and obesity, diet-related chronic diseases and lower life expectancy of Aboriginal and Torres Strait Islander people. A second policy objective described, although to a lesser extent, was one of addressing equity and providing access to healthy food at prices comparable to that of all Australians.

These two objectives were not considered in isolation, with operating costs and commercial viability raised largely, though not solely, by retailers as significant pertinent factors. The cost of food to the store was seen as a significant barrier in implementing health-promoting pricing policies. Participants described the balance required between pricing and profit. Examples were described where storeowners chose to invest their profits in reduced food prices. It was proposed that there is an opportunity to reframe the discourse around profit, by engaging new terms such as ‘retailing for health.’

#### 3.3.4. Decision-Making Criteria

Participants were first asked about the use of six predetermined criteria in policy-making in their context. They were then asked which criteria they considered most important to the process and to identify any gaps in the criteria used. These predetermined criteria were feasibility, sustainability, effectiveness, importance, acceptability and unintended consequences. In describing which criteria were applied, participants described the meaning these criteria had in their context.

Feasibility and sustainability were reported by both retailers and health professionals to be considered in the policy-making processes. A feasible policy was described as one which is achievable in both economic and practical terms, including being a good fit with the existing system, aligning with the available human resource skill set and capacity and the supply of product and infrastructure required to deliver the policy. A sustainable policy was considered to be one which could be continued or scaled-up. A small number of health professionals viewed sustainability as the need for a policy to have appeal to, or be aligned with government policy.

The potential effectiveness of a policy was considered by both retailers and health professionals in policy-making though often with a caveat, such as they ‘assumed’, ‘hoped’, or ‘thought’ a policy might be effective, rather than describing having confidence in a policy’s potential effectiveness. Participants referred to policy as being influenced by the poor population-level health status of Aboriginal and Torres Strait Islander people and current and recommended dietary intake. Rarely however, was research-informed evidence of effectiveness reported to inform policy development.

Participants reported less consideration of the criteria of importance and acceptability. Importance included an assessment of how worthwhile a policy was considered, which was almost solely related to health outcomes. Acceptability related to a policy’s appropriateness to the recipients (i.e., customers) and implementers (i.e., store managers). Both retailers and health professionals reported that it was important to have community buy-in, or that policies be community-driven or at least community-partnered and support capacity enhancement of Aboriginal and Torres Strait Islander storeowners.

Unintended consequences were rarely reported as being considered, though where they were, this was by both retailers and health professionals.

“Unintended consequences—I don’t think we really considered at all. It’s definitely not, if we do, if we drop the price of milk, what will happen next? I don’t think we considered that at all. We, our presumption is always that they (i.e., customers) will continue to spend more money in the store.”

(Interviewee 9, Retailer)

Unintended consequences were perceived as factors that may positively or negatively impact on a health or business outcome. Organization or store brand image and positive or negative publicity were highlighted as emerging unintended consequences that were perceived to impact on business outcomes and recently informed policy development. In relation to health outcomes, one retailer referred to the group of Aboriginal and Torres Strait Islander storeowners he worked with, considering equity across the population in policy development.

Participants were asked to nominate criteria that they considered most important to decision-making and any gaps. All six pre-determined criteria were considered important to decision-making, with the exception that approximately half of the retailers considered assessment of unintended consequences as unnecessary. The order of importance placed on these criteria was generally considered to be context- and policy-specific. No new criteria were identified.

#### 3.3.5. Evidence Informing Decision-Making

There was limited use of research-informed evidence in the processes reported. The three key forms of information used largely originated from the retail sector. Firstly, health professionals, more so than retailers, noted the ‘diffusion of ideas’ or benchmarking as a method which commonly informed local policy. Policy was also informed by food price survey reports and urban store pricing. Secondly, retailers and health professionals referred to the use of store sales in a variety of ways: to conduct retail modelling to inform policy design, to measure retail performance and sales of a targeted product when a pricing policy is implemented, and to provide ongoing monitoring to staff and storeowners in relation to top sellers or targeted products. A reliable point-of-sale system was seen as a requirement for implementing pricing policies, as was the importance of understanding data and disseminating user-friendly reports.

“So a Board that’s not getting nutritional reports back to them, from the store is really not being told enough of the key information. … So that it’s always in their mind and they can see what the store’s doing and then they start to think about their own, well, what if we did this, why can’t we do that, you know. ‘Cause management (i.e., retailers) doesn’t have all the answers.”

(Interviewee 23, Retailer)

The third key information source described was retail, and especially remote retail, industry knowledge. Retailers often described their thinking as influenced by employing the strategies known to work in the retail industry to promote or disincentivize targeted products to shape a health-promoting environment.

### 3.4. Strengthening the Decision-Making Process

#### 3.4.1. Supporting Roles of Decision-Makers

The Aboriginal and Torres Strait Islander and non-Indigenous store committee/board members, retailers and nutritionists, and the relationships between these decision-makers were considered crucial to the process. Opportunities to enhance the current process were proposed: (i) to further support/engage Aboriginal and Torres Strait Islander storeowners, staff and customers in the identification and design of policies, and (ii) to support greater participation of nutritionists, by addressing barriers which included nutritionists either not having the opportunity or not recognizing a role for themselves or their capacity to contribute to policy-making.

Suppliers, whilst not considered to be central to the decision-making process, appeared to have an increasing support role as shown in Table 1. Some suppliers were reported to be supportive having shared values; others, however, were seen as having a poor understanding of the context and promoted unhealthy products even as retailers tried to secure deals on healthy products.

#### 3.4.2. Accessing and Strengthening the Evidence Base

Retailers and health professionals identified three forms of evidence as being potentially useful to the process. The first was accessing research-informed evidence through user-friendly dissemination methods.

“… all the journal articles and big reports and what not are nice, but even people within our (health) organization wanted like almost sound bites, like stories and we needed options in the community and say, ‘This is what’s been done before, here’s the stories and you can choose from these options.’”

(Interviewee 11, Health professional)

The second was further development of locally-informed evidence through improved evaluation and timely feedback to communities. Time and resources were identified as the limiting factor in conducting quality evaluation, not the lack of data. Notably, retailers and health professionals referred to reduced capacity to support activities such as evaluation owing to government funding cuts, resulting in the loss of nutritionist positions in retail and non-government organizations dedicated to working with stores. The third was a better understanding of the factors that drive purchasing decisions, including income and cost of living data and the impact of price on the purchasing of targeted products. Participants sensed that price elasticity of demand varied for different products, that price impacted differently across population groups and that customer response to price is changing. There was also a sense that customers generally may not have all the necessary information available to them in a useable form to make an informed purchasing decision in relation to price.

## 4. Discussion

Health-promoting food pricing policies implemented in very remote Aboriginal and Torres Strait Islander community stores in Australia were dominated by subsidies and subsidy/price increase combinations. These had a small to moderate impact on food prices of fruit, vegetables, bottled water, artificially sweetened and sugar sweetened carbonated beverages, and broadly used ‘healthy/essential’ and ‘unhealthy’ food classifications. Decision-making was a deliberative process, which evaluated policy feasibility and sustainability, though generally lacked incorporation of research-informed evidence.

### 4.1. Designing Health-Promoting Food Pricing Policy

The dominance of subsidies and subsidy/price increases reported in this study is in line with recommendations to support healthier choices in low socioeconomic populations with the subsidy/price increase combination possibly mitigating concerns about the potential regressive nature of taxes [40,41]. The range of products targeted only partially align with the current evidence. The lack of criteria applied to the ‘healthy’ category for example, results in a misalignment with guidelines for good health and a lost opportunity to promote a healthy diet. Targeting artificially sweetened carbonated beverages may not support positive health outcomes as reducing the price of these is unlikely to decrease the consumption of sugar sweetened carbonated beverages [42,43,44,45]. Additionally, there are calls for a greater focus on policy targeting discretionary foods [43,46]. Magnitude of price changes were at best in line with recommendations for modifying purchasing [47]. Equity was the objective of decision-making in some cases, and the magnitude of the price changes went some way to achieving this [48]. The ongoing nature of most policies which are not routinely advertised to customers prevented the use of price as a signal to customers; this was described by participants and supported by others as a significant missed opportunity [49].

Food pricing policies in this context which aim to improve health would be more aligned with research evidence if there was: (i) further targeting of products (e.g., specify healthy foods, foods likely to have a greater response to price changes [43]); (ii) increased magnitude of price change [47,50]; (iii) use of price and price promotion to send a signal to customers, such as through a price increase alone or dynamic, rotating subsidies and promoting the change in price to customers [29,47,49,51]. Policies need to be assessed within the local context and may require new avenues for funding, such as by manufacturers, suppliers and wholesalers, by government or through evaluation of current food pricing policy or funds dispersal.

### 4.2. Enhancing Policy Development Processes

This analysis indicates that the process of decision-making was deliberative [32]. Improved health, and to a lesser extent equity, were key objectives in the decision-making process. These objectives of health and equity inform policy development differently, including the sources of evidence required. Whilst assessment of effectiveness was considered a priority, participant response and the design of current policies, indicates limited use of research-informed evidence. Although consideration of unintended consequences was not universally viewed as important to the process, research-informed evidence would go some way to inform the assessment of this criterion whether it was explicitly included or not. Acceptability and importance were not well-considered criteria, although they were regarded a priority and likely to be best addressed through further engagement with Aboriginal and Torres Strait Islander storeowners and others they elect to involve. Given articulating and communicating problems is a crucial stage in decision-making [32], the processes reported in this context are likely to be improved with further assessment of the criteria, acceptability, importance, effectiveness and unintended consequences of potential policies. The processes were generally focused on a single policy rather than evaluation of a suite of options. They were based on analysis of retail data, informed by an assessment of cost in terms of retail impact though not cost-effectiveness, nor health impact, and limited in terms of robust monitoring and evaluation. Greater incorporation of research-informed evidence into the design of food pricing policies which have an objective of dietary or health improvement, is likely to result in more effective policy, and was called for by study participants [33].

Complex policy with multiple and potentially conflicting objectives, is likely to create tension [32]. There appears to be a shift in the well-documented tension between commercial profit and health outcomes in remote stores [22,23]. Opportunities exist for well-designed health-promoting food pricing policies to be considered within the suite of business practices by storeowners, and precedent has been set for this as described in our study. Currently, retailers are front and center of the decision-making process in remote stores, hence the reliance on retail-focused evidence and criteria in the decision-making process. Current processes offer opportunities to further progress health-promoting policy, such as using the role of benchmarking against other stores and organizations as a potential mechanism for dissemination of good practice. Mechanisms to support decision-makers to access research-informed evidence and to assess acceptability, importance and unintended consequences of policies for the local context could lead to more effective health-promoting policies. This might involve a greater role for Aboriginal and Torres Strait Islander storeowners and nutritionists in decision-making.

### 4.3. Strengths and Limitations

This study has captured the views and experiences of retailers and health professionals across remote Aboriginal and Torres Strait Islander communities in Australia. Effort was made to ensure retailers operating in independent stores were included, though without a census of all stores, this is a more challenging cohort to identify and locate. The resources for this study did not allow for the conduct of interviews in remote communities with Aboriginal and Torres Strait Islander store committee/board members. Interviewing those persons known to work closely with storeowners provided insight into the roles and processes which could be further explored. Participants were invited to contribute where health-promoting food pricing policies were implemented and as such, this is likely to represent the best-case scenario rather than the situation in all remote Aboriginal and Torres Strait Islander community stores. The case considered was food pricing policies, and the process of policy development may be different to that of other health-promoting food policies in stores.

## 5. Conclusions

Remote Aboriginal and Torres Strait Islander community stores provide a crucial setting if health outcomes of their customers are to improve. While owners and operators face major challenges, community ownership provides an opportunity to make a difference to the foods purchased from community stores. The urgency of the situation for Aboriginal and Torres Strait Islander storeowners and those who work to support them is not unlike that of low- and middle-income countries currently leading the way in implementing food-related policies [31,52]. This study identifies opportunities that exist to further shape the store food environment through incorporation of research-informed evidence. In doing so, it offers lessons on how locally-developed and -implemented policies can be formulated to shape other food retail environments for health outcomes. However, addressing equity and positively shaping healthy retail environments should not be a task for storeowners and retailers alone. There is a role for government, manufacturers and wholesalers to work with Aboriginal and Torres Strait Islander storeowners and those who support their efforts, to implement evidence-informed policy to support healthy environments.

## Figures and Tables

**Table 1:** Health-promoting food pricing policies in very remote Aboriginal and Torres Strait Islander community stores in Australia.

Food/Beverage Targeted	Impact on Selling Price	Duration	Administration
***Subsidy***—***Price discount***			
**Fruit and vegetables**—**all fresh**	**Approximately 20% to 25% discount or equal to, to ≤30% of urban retail prices**	**Ongoing**	**Store**
**Fruit and vegetables**—**all fresh, frozen, canned and dried**	**Approximately 20% discount**	**Ongoing**	**Store**
**Water**—**bottled**	**Various, example $0.53, $1.00 and $2.00 for 600 mL**	**Ongoing**	**Store and manufacturer**
Fruit and vegetables—a small range of fresh items	5% to 10% discount or comparable to urban retail prices	Short-term, rotating	Store and supplier
Dairy products—fresh milk, yoghurt and cheese	Approximately 20% discount	Ongoing	Store
Dairy products—low-fat fresh milks	Low-fat milk retailed for the price of full cream milk	Ongoing	Store; Store and manufacturer
Bread—multigrain and wholemeal bread	$1.00 less than white bread	Ongoing	Store
Healthy foods ^1^	n/a	Ongoing and short-term	Store; Store and supplier
Beverages—bottled water and artificially sweetened soft-drink	Various, example bulk packs of bottled water retailing for less than the equivalent volume achieved in single units	Short-term, rotating	Store and manufacturer
***Subsidy—Reward***			
Fruit and vegetables—fresh; fresh, frozen, canned and dried	Various, example a $10 fruit and vegetable gift following a $20 fruit and vegetable purchase	Short-term, including feasibility assessment	Store; Health organization ^2^
Fruit, vegetables, meat ^3^ and bottled water	$25 voucher for health assessment participation	Ongoing	Health organization
***Subsidy—Free***			
Water—chilled via a bubbler outside the store	Free	Ongoing	Store
***Price increase***			
Sugar sweetened carbonated beverages	19% increase	Ongoing	Store
Sugar sweetened carbonated beverages	$0.30 increase per 375 mL can and $1.00 per 1.25 L bottle	Ongoing	Store
***Subsidy/price increase combination***			
**Reduction on healthy foods and increase on unhealthy foods ^1^**	**n/a**	**Ongoing**	**Store**
**Reduction on artificially sweetened carbonated beverages and increase on sugar sweetened carbonated beverages**	**Various ranging from 6% to 22%, and in places a widening gap ^4^**	**Ongoing**	**Store; Stores and manufacturer**

Note: The policies most commonly reported are in bold (i.e., subsidy on fruit and vegetables—all fresh; fruit and vegetables—all fresh, frozen, canned and dried; water—bottled and subsidy/price increase combination on healthy and unhealthy foods and artificially sweetened carbonated and sugar sweetened carbonated beverages). All values are in AUD (AUD1.00 = USD0.77 in 2016). ^1^ Healthy and unhealthy foods were not specified though healthy foods often reported to include commodity groups which were largely though not solely considered to be healthy/core foods such as fruit, vegetables, bread, milk, meat, eggs and infant foods, or items deemed to be essential food items such as tea, sugar and margarine and unhealthy foods often reported to include foods commonly considered to be discretionary foods such as crisps, confectionery, chocolate, biscuits, bakery lines and sugar sweetened beverages. ^2^ Health organization is a local or regional Aboriginal health organization. ^3^ Meat included lean and non-lean cuts of meat. ^4^ It was unclear if this price gap always included a price increase to sugar sweetened carbonated beverages.
